# Patterns of meat and vegetable consumption among community-dwelling adults aged 18 years and older in China

**DOI:** 10.3389/fnut.2025.1706487

**Published:** 2025-11-03

**Authors:** Xiaojing Deng, Hui Cheng, Wenjun Wang, Yalin Hong, Xin Yuan, Huiqing Xu, Yunting Xu, Guofeng Ao, Jian Xu, Yeping Bian, Qing Ye

**Affiliations:** ^1^Geriatric Hospital of Nanjing Medical University, Nanjing, China; ^2^Jiangsu University School of Medicine, Zhenjiang, China; ^3^Nanjing Municipal Center for Disease Control and Prevention, Nanjing, China

**Keywords:** meat, vegetable, consumption pattern, adults, Chinese

## Abstract

**Background:**

Meat and vegetable consumption was each vital for maintaining human health condition. Periodic surveillance and assessment of population-level meat and vegetable consumption are critically important for tailored healthy eating intervention. The primary aim of this study was to investigate the consumption patterns of meat and vegetables among community-dwelling adults in regional China in 2023.

**Methods:**

A cross-sectional survey was conducted in the first year of the post-COVID-19 pandemic in Nanjing Municipality of China. Participants were those residents aged 18 years or above and randomly selected from the whole municipality. The recommendations recently released by the China Nutrition Society in 2022 were used to assess participants’ meat and vegetable consumption level. Logistic regression models were used to identify potential influencing factors of meat and vegetable consumption.

**Results:**

Among the 60,945 participants analyzed, the medians of meat and vegetable consumption were 700.0 g/wk (interquartile range = 375.0, 1,100) and 200.0 g/d (interquartile range = 100.0, 300.0), respectively. Moreover, 13.7% (95%CI = 13.4, 13.9), 18.1% (95%CI = 17.8, 18.5), and 68.2% (95%CI = 67.8, 68.6) of participants consumed meat under, within, and beyond the recommended level, respectively, whereas 71.1% (95%CI = 70.7, 71.4) and 28.9% (95%CI = 28.6, 29.3) consumed vegetables under and reaching the recommended level, respectively. Selected socio-demographic characteristics, lifestyle and behaviors, and chronic conditions were associated with meat and vegetable consumption.

**Conclusion:**

A large proportion of community-dwelling adults consumed meat exceeding the recommended level, whereas a small proportion consumed vegetables reaching the recommended level in regional China in 2023. Moreover, disparities of meat and vegetable consumption existed in socio-demographic characteristics, lifestyle and behaviors, and selected chronic conditions. However, no causality could be inferred due to the nature of the cross-sectional study. For future tailored population-level interventions of healthy eating of meat and vegetables, particular attention should be paid to participants’ socio-demographic characteristics, lifestyle and behaviors, and specific chronic conditions.

## Introduction

Unhealthy eating of meat and vegetables has been identified as a risk factor for developing non-communicable diseases (NCDs), including cardiovascular diseases (CVDs), type 2 diabetes (T2D), and cancers (1–5). From the perspective of public health, population-based interventions of meat and vegetable consumption are vital for the prevention of NCDs (6). For the purpose of initiating tailored intervention strategies of healthy meat and vegetable consumption, it is particularly important to periodically assess population-level patterns of meat and vegetable consumption.

In addition to evaluation of consumption patterns of meat and vegetables, for individualized promotion of healthy eating of meat and vegetables, it is also important to identify potential factors associated with specific food consumption patterns. Usually, in a stable social and living environment, individuals’ food consumption choices are primarily influenced by their socio-economic status, current health conditions, and health knowledge in addition to food supply and price (7–9). Hence, it is necessary to assess meat and vegetable consumption patterns and their associated factors using nutritional epidemiology approaches.

The coronavirus disease-2019 (COVID-19) imposed an unexpected impact on individuals’ meat and vegetable eating behaviors during the pandemic period worldwide, including in China (10–15). Considering that the drivers of food choices were different for residents during and after the COVID-19 emergency, it is meaningful to investigate consumption patterns of food, such as meat and vegetables, and their associated factors in the post-COVID-19 context. Therefore, the present study was developed to investigate consumption patterns of meat and vegetables and their associated factors among community-dwelling adults in regional China in the first year of post-COVID-19.

## Methods

### Study design and participants

This was a broad cross-sectional survey conducted in 2023 in Nanjing municipality, a typical megacity in China. Nanjing had approximately 9.3 million residents and 12 administrative districts in 2020 (16). Of the 12 districts, five were urban and seven were suburban, which was determined using the official definition issued by the China National Bureau of Statistics (17). The primary aims of this study were to investigate: (1) lifestyle (including meat and vegetable consumption) and behavior patterns and (2) common non-communicable diseases (NCDs) (including hypertension, diabetes, chronic obstructive pulmonary disease [COPD], and gastric disorder [gastritis or stomach ulcer]).

Participants who were eligible to take part in the study must be: (1) registered residents in the survey districts, (2) aged 18 years and older, and (3) without cognitive or psychiatric problems. The sample size was estimated at the district level. For each participating district, the sample size was calculated with consideration of the following factors: (1) the lowest prevalence of selected self-reported NCDs (diabetes, hypertension, COPD, and gastric disorder) in local adult residents, 5.4% for self-reported COPD (18); (2) a cross-sectional study design; (3) a multi-stage sampling approach; and (4) an assumed response rate of 85%. Therefore, approximately 11,000 participants would be sufficient for each involved district to warrant an expected statistical power (90%). Consequently, the municipality-level overall sample size was determined to be approximately 55,000 in the context of five districts that would be involved in the study.

**Figure 1:**
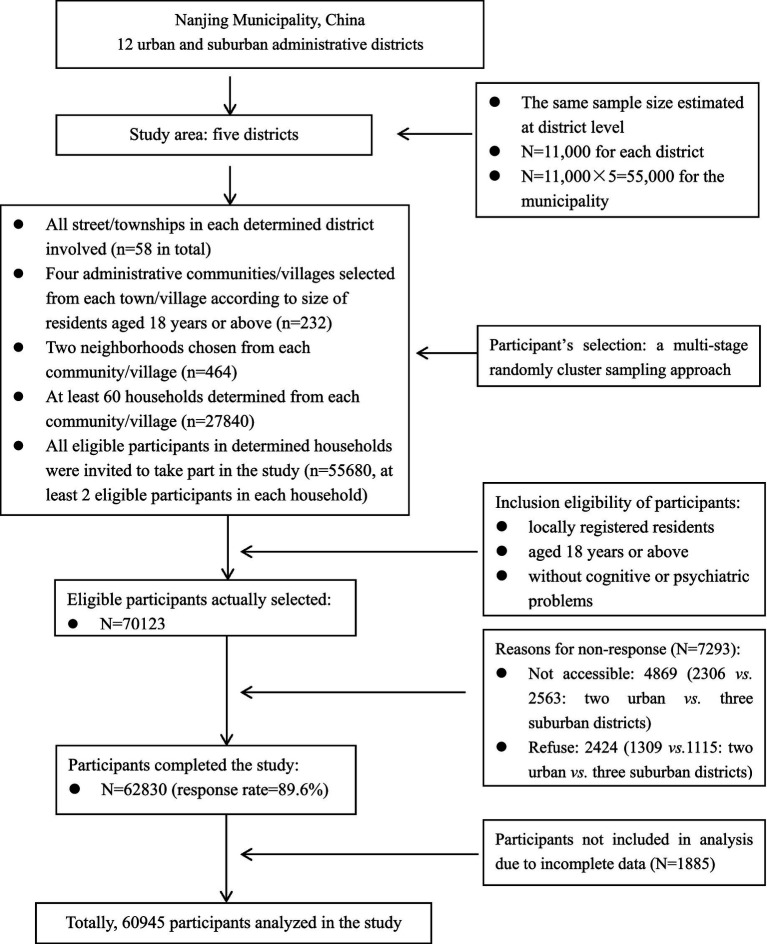
Flowchart of participant’s selection in this study.

A multi-stage sampling method was used to randomly select participants from Nanjing municipality. First, two from five urban and three from seven suburban districts were randomly determined. All streets/townships in these determined districts were included in the survey. Next, four administrative communities/villages were randomly selected from each street/township. Then, two neighborhoods were chosen from each selected community/village. Finally, at least 60 households were determined from each selected neighborhood, and all residents aged 18 years and older in selected households were invited to take part in the survey. It resulted in a total of 55,680 participants being selected, with an assumption of at least two eligible participants within a household. The flowchart of participants’ selection is demonstrated in Figure 1.

Written informed consent was obtained from all participants prior to the survey. The study protocol was approved by the Ethics Committee of Nanjing Municipal Center for Disease Prevention and Control. All the methods used in the study aligned with the Declaration of Helsinki. As only second-hand de-identified data were analyzed in this study, the ethical approval was waived by the Ethics Committee of Geriatric Hospital of Nanjing Medical University.

### Data collection

Data gathered in this study were based on recommendations in the “Scheme of the Chinese chronic non-communicable disease and risk factor surveillance” released by the Chinese Center for Disease Control and Prevention (CCDC), mainly including socio-demographic characteristics, lifestyle and behaviors, common NCDs and associated family histories, and body weight and height (19). Moreover, the standardized interview procedure, questionnaire, specifically validated instruments, and definitions/classifications of variables suggested in the scheme were also used in this study (19). In the local community health service center on the specifically appointed survey date, each participant was interviewed for gathering self-reported information, and anthropometric measures were objectively assessed by research team members (19).

### Study variables

#### Outcome variable

The outcome variable was consumption of meat and vegetables. Red meat, white meat, and vegetable consumption were assessed using a validated Chinese version of the food frequency questionnaire (FFQ) (20). In this FFQ, two sub-items were used to collect consumption information on each food: “(1) how often did you consume this specific food under a typical situation in last year? and (2) on average, how many of the food in LIANG (50 g) did you intake each time?” (20). The Chinese Nutrition Society (CNS) updated the recommended consumption amount of meat (red meat and white meat combined) and vegetables for Chinese adult residents in 2022 (21). The CNS suggested Chinese adults consume 300–500 g of meat per week and a minimum of 300 g fresh vegetables every day in the most recent recommendations (21). Therefore, for the purpose of investigating the consumption patterns, participants were separately categorized into: “under recommendation (consumed meat of <300 g/wk),” “within recommendation (consumed meat of 300–500 g/wk),” or “beyond recommendation (consumed meat of ≥500 g/wk)” based on meat intake recommendation and “under recommendation (consumed vegetables of <300 g/d)” or “reaching recommendation (consumed vegetable of ≥300 g/d)” according to vegetable consumption recommendation in the analysis.

#### Explanatory variables

Several potential factors associated with meat and vegetable intake were analyzed. Participant’s socio-demographic characteristics included age (18–19, 20–29, 30–39, 40–49, 50–59, 60–69, 70–79, or 80 + years old), sex (men or women), residence location (urban or suburban), educational attainment (≤6, 7–12, or ≥13 years of schooling), and marital status (single or having a spouse/partner).

Lifestyle and behaviors, other than meat and vegetable intake, mainly included smoking, drinking, physical activity (PA), and sedentary behavior (SB). The status of smoking (“smokers” or “non-smokers”) and drinking (“drinkers” or “non-drinkers”) was each defined based on classifications recommended by CCDC (19). PA and SB were assessed using the validated Chinese version of the International Physical Activity Questionnaire (IPAQ-CHN) (22). The weekly time of moderate and vigorous PA was measured separately. The sum of moderate PA time and doubled vigorous PA time in the last 7 days was used to classify participants into: “insufficient PA (<150 min/week)” or “sufficient PA (≥150 min/week)” (23). SB level was predicted with daily screen-viewing time and used to categorize participants into: “prolonged SB (≥2 h/day)” or “shortened SB (<2 h/day)” according to adequate SB time recommended specifically for Chinese adults (23).

The definitions of personal histories of diabetes, hypertension, lipid profile, and gastric disorder, as well as family histories of diabetes and hypertension, were adopted from the recommendations by CCDC (19). Participants were also classified as “having diabetes” or “having hypertension” if they self-reported as diagnosed diabetic or hypertensive patients, respectively; otherwise, they were classified as “having no diabetes” or “having no hypertension” (19). Individuals were categorized as “having normal lipid profile” only when they self-reported that the levels of cholesterol, triglyceride, and high/low-density lipoprotein were all normal; otherwise, they were determined as “having abnormal lipid profile” (19). Moreover, participants were determined as “having chronic gastric disorder” if they self-reported that they had been diagnosed with either chronic gastritis or a gastric ulcer; otherwise, they were determined as “having no chronic gastric disorder.” Additionally, participants were classified as “positive family history” if they self-reported at least one parent was a diagnosed diabetic or hypertensive patient; otherwise, they were recorded as “negative family history” (19).

Body weight and height were also assessed based on the standardized procedures recommended by CCDC (19). Briefly, weight and height were each measured twice to the nearest 0.1 kg and 0.01 m, respectively. Using the mean values of weight and height, body mass index (BMI) was calculated as body weight (kg) divided by height squared (m^2^). According to BMI cutoffs recommended for Chinese adults, participants were categorized into: “underweight (BMI < 18.5),” “normal weight (18.5 ≤ BMI < 24.0),” “overweight (24.0 ≤ BMI < 28.0),” or “obesity (BMI ≥ 28.0)” (24).

### Data analysis

First, the consumption levels of meat and vegetables were each tested with a skewed distribution. Using percentage (%) or median (interquartile range, IQR), descriptive analysis was conducted to describe the distribution of meat and vegetable consumption. The differences in meat and vegetable consumption between participants were examined using the chi-square test or Kruskal–Wallis test. Next, with adjustment (where applicable) for age, sex, educational attainment, marital status, body weight, smoking, drinking, PA, SB, diabetes, hypertension, lipid profile, gastric disorder, family histories of diabetes and hypertension, meat intake, and vegetable intake, binary and multinomial logistic regression models were used to compute odds ratios (ORs) and 95% confidence intervals (CIs) to investigate associations of selected factors with vegetable and meat consumption, respectively. The significance level was set as *p* < 0.05 (two-sided). EpiData 3.1 (the EpiData Association 2008, Odense, Denmark) was used to enter data, while SPSS version 20.0 for Windows (SPSS Inc., Chicago, IL, USA) was used for data analysis.

## Results

Totally, 70,123 eligible participants were chosen. There were 7,293 participants (10.4%) who did not respond to the survey (response rate = 89.6%). The main reasons for those who did not respond were “not available” and “refuse.” Among those 62,830 participants taking part in the survey, 1885 (3.0%) submitted incomplete questionnaires and were thus excluded from the analysis. Finally, 60,945 were included in the analysis with complete data. No statistical differences in age and sex were examined between those included in and excluded from the analysis.

Table 1 displays the selected characteristics of participants by residence location. Among the analyzed participants, 3.5 and 1.0% were 18–19 and ≥80 years old, respectively, while 49.4% of participants were men and 38.4% resided in urban areas. Moreover, 42.5% obtained educational attainment of ≥13 years, and 27.3% were single. Additionally, 4.1 and 16.6% were underweight and obese, respectively.

**Table 1:** Selected characteristics of participants in Nanjing municipality in 2023, China (*N* = 60,945).

Characteristics of participants		Participants, % (*n*)			*χ^2^*	*p*-value *
		Overall	Urban	Suburban		
Total		60,945	38.4 (23397)	61.6 (37548)		
Age (years)	18–19	3.5 (2132)	4.0 (945)	3.2 (1187)	498.10	<0.001
	20–29	21.9 (13337)	23.6 (5522)	20.8 (7815)		
	30–39	18.3 (11144)	15.7 (3667)	19.9 (7477)		
	40–49	14.0 (8538)	15.3 (3586)	13.2 (4952)		
	50–59	22.9 (13943)	20.8 (4861)	24.2 (9082)		
	60–69	13.2 (8015)	12.9 (3028)	13.3 (4987)		
	70–79	5.3 (3251)	6.2 (1445)	4.8 (1806)		
	80+	1.0 (585)	1.5 (343)	0.6 (242)		
Sex	Men	49.4 (30090)	50.3 (11770)	48.8 (18320)	13.23	<0.001
	Women	50.6 (30855)	49.7 (11627)	51.2 (19228)		
Educational attainment (years of schooling)	9-	14.1 (8608)	9.7 (2278)	16.9 (6330)	879.25	<0.001
	10–12	43.4 (26437)	41.6 (9743)	44.5 (16694)		
	13+	42.5 (25900)	48.6 (11376)	38.7 (14524)		
Marital status	Single	27.3 (16616)	31.5 (7365)	24.6 (9251)	340.14	<0.001
	Married/having a partner	72.7 (44329)	68.5 (16032)	75.4 (28297)		
Body weight status ^#^	Underweight	4.1 (2483)	4.5 (1044)	3.8 (1439)	245.39	<0.001
	Normal	43.1 (26280)	46.5 (10869)	41.0 (15411)		
	Overweight	36.2 (22055)	34.6 (8106)	22.9 (13949)		
	Obese	16.6 (10127)	14.4 (3378)	18.0 (6749)		

* Chi-square test. # Body weight status was categorized as underweight (BMI<18.5), normal weight (BMI = 18.5, 23.9), overweight (BMI = 24.0, 27.9), or obesity (BMI = ≥28.0) based on recommendations for Chinese adults using the body mass index (BMI).

Table 2 presents the consumption level of meat and vegetables among participants by selected characteristics. The medians of meat and vegetable consumption were 700.0 g/wk (IQR = 375.0, 1,100) and 200.0 g/d (IQR = 100.0, 300.0), respectively, among overall participants. Consumption of meat was significantly different in participants by age, sex, residing location, educational attainment, marital status, body weight, drinking, and smoking, whereas intake of vegetables also differed significantly among participants by each of these factors with the exception of sex.

**Table 2:** Consumption level of meat and vegetables by selected characteristics of participants in Nanjing municipality in 2023, China (*N* = 60,945).

Characteristics of participants		Number of participants, % (n)	Consumption level of meat and vegetables					
			Meat (g/wk)			Vegetables (g/d)		
			Median (25th and 75th IQR)	*H*	*p*-value *	Median (25th and 75th IQR)	*H*	*p*-value *
Total		60,945	700.0 (375.0, 1,100)			200.0 (100.0, 300.0)		
Age (years)	18–19	3.5 (2132)	850.0 (500.0, 1400.0)	1640.73	<0.001	150.0 (78.6, 200.0)	812.32	<0.001
	20–29	21.9 (13337)	850.0 (500.0, 1400.0)			150.0 (100.0, 250.0)		
	30–39	18.3 (11144)	750.0 (450.0, 1250.0)			200.0 (100.0, 300.0)		
	40–49	14.0 (8538)	700.0 (375.0, 1075.0)			200.0 (100.0, 300.0)		
	50–59	22.9 (13943)	675.0 (350.0, 1050.0)			200.0 (100.0, 300.0)		
	60–69	13.2 (8015)	525.0 (300.0, 900.0)			200.0 (100.0, 300.0)		
	70–79	5.3 (3251)	500.0 (300.0, 825.0)			200.0 (100.0, 300.0)		
	80+	1.0 (585)	500.0 (300.0, 725.0)			200.0 (100.0, 300.0)		
Sex	Men	49.4 (30090)	725.0 (425.0, 1250.0)	565.24	<0.001	200.0 (100.0, 300.0)	2.67	0.103
	Women	50.6 (30855)	700.0 (350.0, 1050.0)			200.0 (100.0, 300.0)		
Location	Urban	38.4 (23397)	700.0 (425.0, 1250.0)	270.40	<0.001	196.4 (100.0, 250.0)	262.65	<0.001
	Suburban	61.6 (37548)	700.0 (350.0, 1050.0)			200.0 (100.0, 300.0)		
Educational attainment (years of schooling)	9-	14.1 (8608)	500.0 (300.0, 850.0)	1376.97	<0.001	200.0 (100.0, 300.0)	600.28	<0.001
	10–12	43.4 (26437)	700.0 (350.0, 1050.0)			200.0 (100.0, 300.0)		
	13+	42.5 (25900)	775.0 (500.0, 1350.0)			196.0 (100.0, 250.0)		
Marital status	Single	27.3 (16616)	825.0 (450.0, 1400.0)	515.52	<0.001	150.0 (100.0, 250.0)	533.62	<0.001
	Married/having a partner	72.7 (44329)	700.0 (350.0, 1,050)			200.0 (100.0, 300.0)		
Body weight status ^$^	Underweight	4.1 (2483)	700.0 (400.0, 1250.0)	22.04	<0.001	150.0 (100.0, 250.0)	179.80	<0.001
	Normal	43.1 (26280)	700.0 (375.0, 1100.0)			200.0 (100.0, 300.0)		
	Overweight	36.2 (22055)	700.0 (375.0, 1100.0)			200.0 (100.0, 300.0)		
	Obesity	16.6 (10127)	700.0 (375.0, 1100.0)			200.0 (100.0, 300.0)		
Smoking ^‡^	No	78.1 (47608)	700.0 (363.0, 1050.0)	148.65	<0.001	200.0 (100.0, 300.0)	126.78	<0.001
	Yes	21.9 (13337)	725.0 (425.0, 1250.0)			200.0 (100.0, 300.0)		
Drinking ^¶^	No	71.5 (43584)	700.0 (350.0, 1050.0)	397.88	<0.001	200.0 (100.0, 300.0)	18.44	<0.001
	Yes	28.5 (17361)	750.0 (425.0, 1350.0)			200.0 (100.0, 300.0)		

* Kruskal–Wallis ANOVA test was used to examine the differences in food intake among participants by selected characteristics. $ Body weight status was categorized as underweight (BMI<18.5), normal weight (BMI = 18.5, 23.9), overweight (BMI = 24.0, 27.9), or obesity (BMI ≥ 28.0) based on recommendations for Chinese adults using the body mass index (BMI). ‡ Smoking status was defined based on recommendation by the China National Center for Chronic Disease Control and Prevention. ¶ Drinking status was defined based on recommendation by the China National Center for Chronic Disease Control and Prevention.

Table 3 shows the proportion of participants by consumption recommendations of meat and vegetables. Overall, 13.7% (95%CI = 13.4, 13.9; *n* = 8,320), 18.1% (95%CI = 17.8, 18.5; *n* = 11,056), and 68.2% (95%CI = 67.8, 68.6; *n* = 41,569) of participants consumed meat under, within, and beyond the recommended level, respectively, whereas 71.1% (95%CI = 70.7, 71.4; *n* = 43,314) and 28.9% (95%CI = 28.6, 29.3; *n* = 17,631) consumed vegetables under and reaching the recommended level, respectively. Additionally, the proportions of participants who consumed different levels of meat and vegetables varied significantly across age, sex, residence location, education, marital status, body weight, smoking, drinking, PA, SB, diabetes, hypertension, lipid profile, and gastric disorder, respectively.

**Table 3:** Selected characteristics of participants by meat and vegetable consumption recommendation in Nanjing municipality in 2023, China.

Characteristics of participants		Number of participants with each category	Participants by consumption recommendation of meat and vegetables, % (n)								
			Meat ^*^					Vegetables (consumption level reached) ^*^			
			﹤ 300 g/wk	300-500 g/wk	≥ 500 g/wk	*χ^2^*	p-value ^#^	﹤ 300 g/d	≥ 300 g/d	*χ^2^*	p-value ^#^
Overall		60,945	13.7 (8320)	18.1 (11056)	68.2 (41569)			71.1 (43314)	28.9 (17631)		
Age (years)	18–19	2,132	10.5 (224)	13.8 (295)	75.7 (1613)	1673.25	<0.001	79.9 (1704)	20.1 (428)	647.23	<0.001
	20–29	13,337	9.2 (1228)	14.2 (1888)	76.6 (10221)			77.8 (10372)	22.2 (2965)		
	30–39	11,144	10.3 (1147)	15.4 (1719)	74.3 (8278)			72.2 (8046)	27.8 (3098)		
	40–49	8,538	12.7 (1085)	18.7 (1598)	68.6 (5855)			70.4 (6014)	29.6 (2524)		
	50–59	13,943	15.7 (2194)	20.9 (2914)	63.4 (8835)			66.3 (6251)	33.7 (4692)		
	60–69	8,015	19.0 (1525)	21.7 (1739)	59.3 (4751)			66.7 (5343)	33.3 (2672)		
	70–79	3,251	23.9 (778)	23.7 (769)	52.4 (1704)			66.2 (2152)	33.8 (1099)		
	80+	585	23.8 (139)	22.9 (134)	53.3 (312)			73.8 (432)	26.2 (153)		
Sex	Men	30,090	11.4 (3417)	16.5 (4975)	72.1 (21698)	446.82	<0.001	70.7 (21286)	29.3 (8804)	3.14	0.076
	Women	30,855	15.9 (4903)	19.7 (6081)	64.4 (19871)			71.4 (22028)	28.6 (8827)		
Location	Urban	23,397	10.7 (2514)	16.7 (3918)	72.5 (16965)	378.82	<0.001	75.1 (17563)	24.9 (5834)	294.73	<0.001
	Suburban	37,548	15.5 (5806)	19.0 (7138)	65.5 (24604)			68.6 (25751)	31.4 (11797)		
Educational attainment (years of schooling)	9-	8,608	23.9 (2058)	22.5 (1941)	53.5 (4609)	1800.91	<0.001	65.5 (5637)	34.5 (2971)	539.46	<0.001
	10–12	26,437	14.5 (3833)	19.8 (5231)	65.7 (17373)			68.1 (18011)	31.9 (8426)		
	13+	25,900	9.4 (2429)	15.0 (3884)	75.6 (19587)			75.9 (19666)	24.1 (6234)		
Marital status	Single	16,616	11.0 (1825)	14.6 (2434)	74.4 (12357)	399.85	<0.001	77.4 (12858)	22.6 (3758)	442.76	<0.001
	Married/having a partner	44,329	14.7 (6495)	19.5 (8622)	65.9 (29212)			68.7 (30456)	31.3 (13873)		
Body weight status ^†^	Underweight	2,483	12.8 (319)	16.2 (402)	71.0 (1762)	34.97	<0.001	78.0 (1937)	22.0 (546)	151.86	<0.001
	Normal	26,280	13.3 (3497)	18.1 (4745)	68.6 (18038)			72.7 (19118)	27.3 (7162)		
	Overweight	22,055	13.9 (3069)	19.0 (4184)	67.1 (14802)			69.3 (15292)	30.7 (6763)		
	Obese	10,127	14.2 (1435)	17.0 (1725)	68.8 (6967)			68.8 (6967)	31.2 (3160)		
Smoking ^$^	No	47,608	14.1 (6719)	18.5 (8808)	67.4 (32081)	71.03	<0.001	72.1 (34326)	27.9 (13282)	112.41	<0.001
	Yes	13,337	12.0 (1601)	16.9 (2248)	71.1 (9488)			67.4 (8988)	32.6 (4349)		
Drinking ^$^	No	43,584	14.6 (6364)	19.0 (8278)	66.4 (28942)	235.28	<0.001	71.5 (31178)	28.5 (12406)	16.08	<0.001
	Yes	17,361	11.3 (1956)	16.0 (2778)	72.7 (12627)			69.9 (12136)	30.1 (5225)		
Physical activity (leisure time) ^†^	Insufficient (<150 min/wk)	49,027	14.4 (7070)	18.5 (9081)	67.1 (32876)	177.36	<0.001	71.8 (35207)	28.2 (13820)	66.92	<0.001
	Sufficient (≥150 min/wk)	11,918	10.5 (1250)	16.6 (1975)	72.9 (8693)			68.0 (8107)	32.0 (3811)		
Sedentary behavior ^†^	Prolonged (≥2 h/d)	55,300	20.3 (1147)	17.8 (9863)	69.2 (38264)	318.52	<0.001	68.4 (3860)	28.7 (15846)	21.93	<0.001
	Shortened (<2 h/d)	5,645	13.0 (7172)	21.1 (1193)	58.5 (3305)			71.3 (39453)	31.6 (1785)		
Gastritis ^☆^	No	52,629	13.3 (7024)	18.0 (9458)	68.7 (36147)	45.26	<0.001	71.8 (37777)	28.2 (14852)	94.35	<0.001
	Yes	8,316	15.6 (1296)	19.2 (1598)	65.2 (5422)			66.6 (5537)	33.4 (2779)		
Diabetes ^¶^	No	56,580	13.2 (7449)	18.0 (10162)	68.9 (38968)	201.31	<0.001	71.5 (40440)	28.5 (16140)	63.06	<0.001
	Yes	4,365	19.9 (870)	20.5 (894)	59.6 (2601)			65.8 (2873)	34.2 (1492)		
Hypertension ^¶^	No	49,875	12.7 (6336)	17.5 (8707)	69.8 (34831)	357.64	<0.001	72.3 (36045)	27.7 (13829)	192.93	<0.001
	Yes	11,070	17.9 (1983)	21.2 (2349)	60.9 (6738)			65.7 (7268)	34.3 (3802)		
Lipid profile ^¶^	Abnormal	56,051	13.4 (7492)	18.0 (10107)	68.6 (38451)	61.49	<0.001	71.5 (40100)	28.5 (15950)	75.98	<0.001
	Normal	4,894	16.9 (827)	19.4 (949)	63.7 (3118)			65.7 (3213)	34.3 (1681)		

* Meat was defined as either red meat or white meat and classified as "lower than recommended level (<300 g/wk)", "between recommended level (300–500 g/wk)" or "above recommended level (≥500 g/wk)", while vegetable consumption was categorized as "lower than recommendation (<300 g/d)" or "recommendation reached (≥300 g/d)" according to recommendations by Chinese Nutrition Society in 2022. # Chi-square test. † Body weight status was categorized as underweight (BMI < 18.5), normal weight (BMI = 18.5, 23.9), overweight (BMI = 24.0, 27.9), or obesity (BMI ≥ 28.0) based on recommendations for Chinese adults using body mass index (BMI). $ Smoking and drinking status were each defined based on recommendation by China National Center for Chronic Disease Control and Prevention. ‡ Physical activity in last 7 days and daily sedentary behavior time were measured using the International Physical Activity Questionnaire, and classified as "sufficient" or "insufficient", and "shortened" or "prolonged", respectively, based on specific recommendations for Chinese adults. ☆ Gastritis refers to chronic gastritis, which was self-reported by participants. ¶ Status of diabetes, hypertension and lipid profile were each self-reported by participants.

Table 4 demonstrates the odds for participants with different characteristics to consume meat and vegetables by recommendation level, respectively. After mutual adjustment for potential associated factors, women, suburban residents, participants with shortened SB, or hypertensive adults were less likely to meet the recommended consumption level of meat, whereas participants with a higher level of education, spouse/partner, or sufficient PA were more likely to meet the recommended level of meat consumption. Moreover, older individuals, women, suburban residents, and individuals with shortened SB or diabetes were at lower odds to consume meat beyond the recommended level, whereas participants with higher educational levels, spouses/partners, drinking habits, or sufficient PA were at higher odds to consume meat beyond the recommended level of meat. Additionally, age, sex, residence location, education, marital status, body weight, smoking, PA, hypertension, lipid profile, and gastric disorder were each significantly associated with the odds for participants to meet the recommended level of vegetable consumption.

**Table 4:** Association of selected characteristics with likelihood of meeting recommendation of meat and vegetable consumption among participants in Nanjing municipality in 2023, China.

Characteristics of participants		Number of participants with each category	OR (95%CI) for participants to consume different level of meat and vegetables *					
			Meat ^#^				Vegetables	
			Intake amount between recommended levels (300-500 g/wk)		Intake amount exceeding recommended upper level (≥500 g//wk)		Intake amount reaching recommendation (≥300 g/d)	
			Model 1 ^$^	Model 2 ^†^	Model 1 ^$^	Model 2 ^†^	Model 1 ^$^	Model 2 ^†^
Overall		60,945						
Age (years)	18–19	2,132	1	1	1	1	1	1
	20–29	13,337	1.17 (0.97, 1.41)	1.10 (0.91, 1.34)	1.16 (0.99, 1.35)	1.01 (0.86, 1.18)	1.14 (1.02, 1.28)	**1.17 (1.05, 1.32)**
	30–39	11,144	1.14 (0.94, 1.38)	1.00 (0.81, 1.23)	1.002 (0.86, 1.17)	0.88 (0.74, 1.05)	1.53 (1.37, 1.72)	**1.35 (1.19, 1.53)**
	40–49	8,538	1.12 (0.93, 1.35)	1.01 (0.82, 1.26)	0.75 (0.64, 0.87)	**0.72 (0.61, 0.86)**	1.67 (1.49, 1.88)	**1.38 (1.21, 1.57)**
	50–59	13,943	1.01 (0.84, 1.21)	1.01 (0.82, 1.24)	0.56 (0.48, 0.65)	**0.65 (0.55, 0.78)**	2.02 (1.81, 2.26)	**1.52 (1.34, 1.73)**
	60–69	8,015	0.87 (0.72, 1.04)	0.88 (0.71, 1.09)	0.43 (0.37, 0.50)	**0.53 (0.44, 0.63)**	1.99 (1.77, 2.24)	**1.50 (1.31, 1.72)**
	70–79	3,251	0.75 (0.61, 0.92)	0.81 (0.65, 1.02)	0.30 (0.26, 0.36)	**0.39 (0.32, 0.48)**	2.03 (1.79, 2.31)	**1.60 (1.39, 1.86)**
	80+	585	0.73 (0.55, 0.98)	0.79 (0.58, 1.08)	0.31 (0.24, 0.40)	**0.39 (0.30, 0.51)**	1.41 (1.14, 1.74)	1.24 (0.99, 1.54)
Sex	Men	30,090	1	1	1	1	1	1
	Women	30,855	0.85 (0.80, 0.90)	**0.88 (0.82, 0.94)**	0.64 (0.61, 0.67)	**0.73 (0.68, 0.77)**	0.97 (0.94, 1.003)	**1.05 (1.01, 1.10)**
Location	Urban	23,397	1	1	1	1	1	1
	Suburban	37,548	0.79 (0.74, 0.84)	**0.81 (0.76, 0.86)**	0.63 (0.60, 0.66)	**0.63 (0.60, 0.67)**	1.38 (1.33, 1.43)	**1.33 (1.28, 1.38)**
Educational attainment (years of schooling)	9-	8,608	1	1	1	1	1	1
	10–12	26,437	1.45 (1.34, 1.56)	**1.30 (1.19, 1.40)**	2.02 (1.90, 2.16)	**1.47 (1.38, 1.58)**	0.89 (0.84, 0.93)	**0.91 (0.86, 0.96)**
	13+	25,900	1.70 (1.57, 1.84)	**1.49 (1.34, 1.65)**	3.60 (3.37, 3.85)	**2.10 (1.93, 2.30)**	0.60 (0.57, 0.63)	**0.72 (0.68, 0.77)**
Marital status	Single	16,616	1	1	1	1	1	1
	Married/having a partner	44,329	0.995 (0.93, 1.07)	**1.18 (1.07, 1.30)**	0.66 (0.63, 0.70)	**1.16 (1.06, 1.26)**	1.56 (1.50, 1.63)	**1.16 (1.09, 1.23)**
Body weight status ^‡^	Underweight	2,483	1	1	1	1	1	1
	Normal	26,280	1.08 (0.92, 1.26)	1.11 (0.95, 1.30)	0.93 (0.82, 1.06)	1.10 (0.96, 1.25)	1.33 (1.20, 1.47)	**1.11 (1.01, 1.23)**
	Overweight	22,055	1.08 (0.93, 1.26)	1.15 (0.98, 1.36)	0.87 (0.77, 0.99)	1.13 (0.99, 1.29)	1.57 (1.42, 1.73)	**1.16 (1.05, 1.29)**
	Obese	10,127	0.95 (0.81, 1.12)	1.02 (0.86, 1.21)	0.88 (0.77, 1.003)	1.14 (0.99, 1.31)	1.61 (1.45, 1.79)	**1.19 (1.07, 1.32)**
Smoking ^◇^	No	47,608	1	1	1	1	1	1
	Yes	13,337	1.07 (0.997, 1.15)	1.00 (0.91, 1.09)	1.24 (1.17, 1.32)	1.07 (0.99, 1.15)	1.25 (1.20, 1.30)	**1.12 (1.06, 1.18)**
Drinking ^◇^	No	43,584	1	1	1	1	1	1
	Yes	17,361	1.09 (1.02, 1.16)	1.00 (0.93, 1.08)	1.42 (1.34, 1.50)	**1.14 (1.07, 1.21)**	1.08 (1.04, 1.13)	1.01 (0.96, 1.05)
Physical activity (leisure time)	Insufficient	20,083	1	1	1	1	1	1
	Sufficient	40,862	1.23 (1.14, 1.33)	**1.18 (1.09, 1.28)**	1.50 (1.40, 1.60)	**1.32 (1.23, 1.41)**	1.20 (1.15, 1.25)	**1.24 (1.19, 1.30)**
Sedentary behavior	Prolonged≥2 h/d	55,300	1	1	1	1	1	1
	Shortened <2 h/d	5,645	0.76 (0.69, 0.83)	**0.84 (0.77, 0.92)**	0.54 (0.50, 0.58)	**0.75 (0.70, 0.81)**	1.15 (1.09, 1.22)	0.98 (0.92, 1.04)
Gastritis^☆^	No		1	1	1	**1**		
	Yes		0.92 (0.85, 0.99)	0.97 (0.90, 1.06)	0.81 (0.76, 0.87)	0.97 (0.90, 1.04)	1.28 (1.22, 1.34)	**1.15 (1.09, 1.21)**
Diabetes ^¶^	No	56,580	1	1	1	1	1	1
	Yes	4,365	0.75 (0.68, 0.83)	0.93 (0.83, 1.03)	0.57 (0.53, 0.62)	**0.82 (0.75, 0.89)**	1.30 (1.22, 1.39)	1.03 (0.96, 1.11)
							
Hypertension ^¶^	No	49,875	1	1	1	1	1	1
	Yes	11,070	0.86 (0.80, 0.92)	**0.83 (0.74, 0.92)**	0.62 (0.58, 0.65)	0.94 (0.88, 1.01)	1.36 (1.31, 1.43)	**1.08 (1.02, 1.13)**
Lipid profile ^¶^	Abnormal	56,051	1	1	1	1	1	1
	Normal	4,894	0.85 (0.77, 0.94)	0.92 (0.83, 1.03)	0.74 (0.68, 0.80)	0.97 (0.89, 1.06)	1.32 (1.24, 1.40)	**1.07 (1.01, 1.15)**

* OR (odds ratio) and CI (confidence interval) were calculated with “intake amount less than recommended level” as the reference. # Meat was defined as either red meat or white meat and classified as ‘lower than recommended level,” “between recommended level,” or “above recommended level,” while vegetable consumption was categorized as “lower than recommendation” or “recommendation reached” according to recommendations by the Chinese Nutrition Society in 2022. $ Model 1 was univariate logistic regression analysis. † Model 2 was a multivariate logistic regression analysis with mutual adjustment (where applicable) for age, sex, education, marital status, body weight status, smoking, drinking, physical activity, sedentary behavior, self-reported diabetes, self-reported hypertension, self-reported abnormal lipid profile, family history of hypertension, family history of diabetes, meat intake, and vegetable intake in multivariate logistics regression models. ‡ Body weight status was categorized as underweight (BMI<18.5), normal weight (BMI = 18.5, 23.9), overweight (BMI = 24.0, 27.9), or obesity (BMI≥28.0) based on recommendations for Chinese adults using the body mass index (BMI). ◇ Smoking and drinking status were each defined based on recommendations by the China National Center for Chronic Disease Control and Prevention. ☆ Gastritis refers to chronic gastritis, which was self-reported by participants. ¶ Diabetes, hypertension, and lipid profile were self-reported by participants. The bold values indicate “statistically significant”.

## Discussion

In this community-based nutritional epidemiological study, the primary aims were to investigate the patterns of meat and vegetable consumption among adults aged 18 years and older in regional China in the first year of the post-COVID-19 epidemic. It was observed that: (1) the median consumption levels of meat and vegetables were 700.0 g/wk and 200.0 g/d, respectively, and (2) the proportions of participants who consumed within-and beyond-recommended levels of meat were 18.1 and 62.8%, respectively, whereas 71.9% consumed less vegetables, and only 28.9% met the recommended level of vegetable consumption among overall participants. Additionally, several socio-demographic characteristics, lifestyle and behaviors, and chronic conditions were significantly associated with the odds for participants to meet the recommended level of meat and vegetable consumption.

This is the first survey investigating the consumption patterns of meat and vegetables among adults aged 18 years and older in the post-COVID-19 context in China. Thus, there are no similar studies available for us to make a comparison with ours. However, it is still of interest to make comparisons between the findings in our study and those conducted prior to COVID-19 in China. A nation-wide epidemiological study on nutrition and health, the China Nutrition and Health Survey (CNHS), was initiated in 1989 in China (25). In the most recent CNHS studies conducted in 2015 and 2018, mean values of meat intake were 658.0 g/wk among residents aged 18–59 years and 765.3 g/wk among participants aged 18–64 years, respectively (26, 27). In our study, the mean value of meat intake (893.8 g/wk) among individuals aged 18 years and older was higher than those investigated in the two national-level surveys prior to COVID-19, although participants of different ages were analyzed in these three studies.

As for vegetable intake, interestingly, different mean values were reported from two nation-wide surveys conducted in the same year of 2018 in China (27, 28). CNHS-2018 reported that the average amount of vegetable intake was 261.1 g/d among Chinese adults aged 18–64 years, whereas another nation-level survey documented that the mean value of vegetable intake was 369.1 g/d among adults aged 18 years and older (27, 28). In our study, the average intake level of vegetables was 209.9 g/d among participants aged 18 years and above, which was lower than those recorded in the two nation-wide studies prior to the COVID-19 epidemic (27, 28).

Regarding the proportion of participants meeting recommended intake levels in CNHS-2018, 62.6% of participants consumed meat beyond the recommended level, whereas 60.0% consumed vegetables under the recommended level (27). In our study, 68.2 and 71.1% of participants consumed meat above the recommended level and vegetables under the recommended level, respectively. The differences in the consumption amount of meat and vegetables and the proportion of participants reaching intake recommendations between previous studies and ours may be explained by the fact that participants of different ages and residence regions were involved, and data were gathered in different years (before and after the COVID-19 epidemic).

Typically, under a stable social and living context, the main driving factors of residents’ food consumption choices were food supply, price, participant’s socioeconomic characteristics, health conditions, health knowledge, and related behaviors (7–9). After the COVID-19 pandemic, the social life and living environment returned to the normal state (including food supply and price) and remained relatively stable. Therefore, it was plausible that participants’ food choices might primarily depend on the aforementioned personal characteristic drivers of food choices (7–9). This may partially explain the factors associated with meat and vegetable consumption identified in this study—participants’ socio-demographic attributes, lifestyle and behaviors, and chronic conditions.

The present study has public health implications. Periodical surveillance of dietary patterns is critical for healthy eating intervention and subsequent prevention of eating-related NCDs. Emergency events such as the COVID-19 epidemic can impose unexpected impacts on food supply and residents’ food choices and thus exert influence on individuals’ eating behaviors. In the post-COVID-19 context, where social life returned to normal as it was before the disease outbreak, it is particularly important to investigate residents’ eating behaviors for the purpose of initiating precision intervention programs against dietary-related NCDs. For example, for younger adults and men, they each should be encouraged to eat more vegetables and less meat, while single or physically inactive individuals should be educated to eat more vegetables. The identified patterns of eating behaviors among residents can be used as the reference data for long-term comparison and assessment of population-level food intake, which can inform policymakers to optimize the surveillance system of dietary-related NCDs in China.

Several strengths are worthy of being mentioned in the study. First, participants were randomly determined and representative of the general residents aged 18 years and older in the whole municipality of Nanjing. Second, validated instruments and standardized procedures of information collection were used, warranting that data would be comparable. Third, the most recent consumption recommendations were used to evaluate residents’ meat and vegetable consumption level. Finally, as the first investigation in the context of post-COVID-19, the findings are meaningful to inform population-level dietary-related NCD prevention in China.

Some limitations should also be addressed. First, due to the nature of cross-sectional survey, no causality could be inferred for the associations of lifestyle/behaviors and chronic conditions in this study. Second, although validated instruments were used, information on meat and vegetable consumption was self-reported by participants. It was documented that Chinese adults would under-report their consumption of meat and vegetables even using a validated FFQ (29), which implied that the intake levels of meat and vegetables might be under-estimated in the present study. Consequently, the proportion of participants who met intake recommendations of meat and vegetables would also be under-estimated in this study. Therefore, such a recall bias should be considered when interpreting the study findings. Third, only four selected NCDs—diabetes, hypertension, abnormal lipids, and gastric disorder—were adjusted for in the multivariate analysis. Other NCDs such as cancers and cardiovascular diseases that might influence meat and vegetable consumption were not controlled for in the analysis due to a lack of data, potentially leading to an over-estimated statistical power. Fourth, data on NCDs were also self-reported by participants, which might under-estimate the cases due to recall bias. It is encouraged to gather information on NCDs via self-report together with confirmation using medical records in future surveys. Fifth, it is known that a food source may affect its nutritional value and its consumer health. However, due to a lack of data, we could not include the source of meat and vegetables in the analysis. Sixth, the NCDs analyzed were also self-reported, which might under-estimate the prevalent cases in the study. This might imply that cases of NCDs were under-adjusted for in the multivariate analysis. Finally, notably, consumption patterns of meat and vegetables and associated factors identified in this study were from a single city, implying that they could not be extrapolated to other regions in China. In the future, periodical investigations of meat, vegetables, and other main types of food should be conducted within different regions of China, possibly using the 24-h dietary recall or diary approach, to evaluate the long-term consumption trends for tailored population-level promotion of healthy eating among residents.

In conclusion, among residents aged 18 years and older in regional China, a large proportion consumed meat exceeding the recommended level, whereas a small proportion consumed vegetables reaching the recommended level. Moreover, socio-demographic characteristics, lifestyle and behaviors, and selected chronic conditions were associated with meat and vegetable consumption. This study has public health implications, suggesting that particular attention should be paid to participants’ socio-demographic characteristics, lifestyle and behaviors, and specific chronic conditions in tailored population-level interventions of healthy eating of meat and vegetables in the future.

## Data Availability

The raw data supporting the conclusions of this article will be made available by the authors, without undue reservation.
